# Comorbidity of Infectious Diseases and Anxiety Disorders in Adults and Its Association with Quality of Life: A Community Study

**DOI:** 10.3389/fpubh.2014.00080

**Published:** 2014-07-14

**Authors:** Cornelia Witthauer, Andrew T. Gloster, Andrea Hans Meyer, Renee D. Goodwin, Roselind Lieb

**Affiliations:** ^1^Department of Psychology, Division of Clinical Psychology and Epidemiology, University of Basel, Basel, Switzerland; ^2^Department of Psychology, Queens College and The Graduate Center, City University of New York, New York, NY, USA; ^3^Department of Epidemiology, Mailman School of Public Health, Columbia University, New York, NY, USA

**Keywords:** representative survey, anxiety disorder, comorbidity, infectious diseases, quality of life

## Abstract

**Objective:** Infectious diseases and anxiety disorders are common and both are associated with substantial burden to individual, families, and society. A better understanding of their association may be helpful in explicating possible etiological mechanisms related to both. The goal of the current study was to investigate the relationship between specific infectious diseases and anxiety disorders among adults in the community, and to examine whether the co-occurrence of the two is associated with poorer quality of life compared to subjects with one or neither condition.

**Methods:** We used data from the 1998 German Mental Health survey with 4181 subjects aged 18–65. Various infectious diseases (lifetime) and health-related quality of life were assessed via self-report questionnaires and anxiety disorders (past 12-months) were diagnosed using M-CIDI interviews. Logistic regression analyses were used to evaluate the association between infectious diseases and anxiety disorders; a linear model adjusted for sex was used to examine whether comorbidity of infectious diseases and anxiety disorders was associated with quality of life.

**Results:** Whooping cough [odds ratio (OR) = 1.69, 95% confidence intervals (CI) = 1.36–2.09], scarlet fever (OR = 1.31, 95% CI = 1.02–1.68), and diphtheria (OR = 1.79, 95% CI = 1.21–2.64) were associated with increased prevalence of any anxiety disorder. Subjects with both infectious diseases and anxiety disorders reported lower levels of both mental and physical quality of life, compared with subjects with only one or neither condition.

**Conclusion:** Extending prior research, this study suggests a relationship between specific infectious diseases and anxiety disorders in an adult community sample. Research targeting etiological mechanisms related to the interplay between infectious diseases and anxiety disorders is warranted.

## Introduction

Approximately 26% of the total global burden of disease is attributable to infectious diseases (1). Studies have also revealed that mental disorders are a leading contributor to the total all cause burden of disease worldwide (2, 3). Such rates render the refined understanding, treatment, and prevention of both infectious disease and mental disorders as important public health goals. Toward this end, it is crucial to understand etiological pathways involved in disease and associated burden. Importantly, increasing evidence points to associations between some infectious diseases and mental disorders that may hint at overlapping etiologic pathways.

Major depressive disorder is associated with streptococcal infections (4). Further, severe infections such as hepatitis infections or sepsis infections are associated with subsequent mood disorder diagnosis in a longitudinal study (5). In addition, studies have shown that pre-natal exposure to viruses increases the offsprings risk of unipolar affective disorder (6). Due to the association of infectious diseases and affective disorders, there have been efforts to detect potential etiological mechanisms related to both infectious diseases and affective disorders (7, 8).

While much is known about infectious disease and mood disorders, relatively little has been done to investigating the possibly link between infectious disease and anxiety disorders. Some research findings suggest that infectious diseases could be related to the development of specific anxiety disorders. Among infectious diseases, childhood group A streptococcal infections appear to be associated with obsessive–compulsive disorder and/or tic disorders (pediatric autoimmune neuropsychiatric disorders associated with streptococcal infection or PANDAS) (9) based on the results of several studies. Other studies have found increased prevalence rates of posttraumatic stress disorder in HIV positive subjects across different demographic, cultural, and socioeconomic backgrounds (10). Additionally, anxiety disorders are associated with inflammatory markers such as C-reactive protein in men (11) and were found to be associated with having experienced a common cold during the last 12 months compared to subjects without (12). Furthermore, it was found that in older subjects with cytomegalovirus (CMV) antibodies, individuals with higher CMV specific antibody titers were more likely to be anxious than subject with lower CMV antibodies (13). A cross-sectional study looking at the association of infections in the first year of life (reported by the parents) and anxiety disorders found that there is an association between having any infection during the first year of life and anxiety disorders (14). However, no differentiation was made between different types of infections in this study. Additionally, very small cell sizes prohibited more informative analyses (14). No prior study has examined the relationship between anxiety disorders and specific infectious disease among adults in the community.

Additionally, research has shown that the comorbidity of anxiety disorders and physical health problems such as chronic physical diseases is related to a decrease in health-related quality of life (15). To date, no study has investigated whether the comorbidity between infectious diseases and anxiety disorders is associated with an excess loss of health-related quality of life.

The goal of the current study was to investigate the relationship between infectious diseases and anxiety disorders in a larger cross-sectional sample of adults in the community. We also investigated whether comorbidity of anxiety disorders and infectious disease is associated with an excess loss of health-related quality of life.

## Materials and Methods

### Design and sample

Data were drawn from the German Health Interview and Examination Survey and its Mental Health Supplement (GHS-MHS) conducted in 1997. The German Health Survey (GHS) was the first nationwide cross-sectional study for medical and social assessments in Germany, commissioned by the German Ministry of Science, Research and Education, and the Robert Koch Institute and authorized by the relevant institutional review board and ethics committee. The aim of the core study was the assessment of sociodemographic characteristics, physical diseases, impairments, and healthcare utilization in a representative community sample of 7124 subjects aged 18–79 (Overall Response Rate: 61.5%). It was a stratified, randomized sample from 113 communities throughout Germany with 130 sampling units (sampling steps: 1: selection of communities, 2: selection of sampling units, 3: selection of inhabitants) (16, 17). To handle the stratified sampling design the data were weighted and confidence intervals (CI) were calculated by the Huber–White sandwich method to account for the weighting scheme as well as the stratified sampling design (16).

For the assessment of mental disorders in the GHS-MHS a two-stage design was used: The first stage entailed the administration of a 12-item screening questionnaire for mental disorders at the end of the medical examination of the core survey (CID-S) (18). The second stage involved the administration of a structured psychopathological interview, the Munich Composite International Diagnostic Interview (DIA-X/M-CIDI) to all core survey respondents who had been screened positive for a mental disorder and to a random sample of 50% who screened negative (18). This subsample of the GHS built the sample of the Mental Health Supplement and included 4181 subjects aged 18–65 years. The conditional response rate (i.e., subjects who completed the M-CIDI interview) was 87.6%. All participants gave their informed consent. Further description of aims, design, and methods as well as sociodemographic characteristics of the whole GHS-MHS sample can be found elsewhere (16).

### Measures

#### Mental disorders

For the diagnostic assessments, a modified version of the fully structured interview DIA-X/M-CIDI was used (19). The questions cover DSM-IV and ICD-10 criteria. The DIA-X interview enables the assessments of symptoms, syndromes and onset, duration, and severity. The interview was conducted by trained psychologists and physicians (20). The DIA-X/M-CIDI diagnostic algorithms were used to obtain diagnostic findings reported in this paper (21). The test–retest reliability of the DIA-X/M-CIDI was substantial (kappa values ranging between 0.56 and 0.81) (20) and the sensitivity of the DIA-X/M-CIDI diagnoses ranges from 87.5 to 100%; and their specificity from 71.2 to 100% (22). Analyses revealed that the validity of the full diagnoses ranges from moderate to excellent when compared to diagnoses administered from independent treating physicians in a sample of randomly chosen patients (22).

The present study used the following 12-months DSM-IV mental disorders: agoraphobia, social phobia, specific phobia (animal, natural environment, blood–injection–injury, situational type), panic disorder, generalized anxiety disorder, and obsessive–compulsive disorder. We also included panic attacks during the last 12 months.

#### Infectious diseases

Subjects were queried via a paper–pencil questionnaire: “which of the following infectious diseases did you have during your lifetime: diphtheria, whooping cough, measles, mumps, rubella, chicken pox, scarlet fever, tuberculosis, dysentery, or typhus?”

#### Quality of life

We used the German version (23) of the well-validated (24–26) SF-36 quality of life questionnaire. The SF-36 assesses health-related quality in eight dimensions during the past 30 days (physical functioning, social functioning, role limitations due to physical problems, bodily pain, mental health, role limitations due to emotional problems, vitality, and general health). Principal component analysis revealed two robust factor dimensions of physical and mental health: the Physical Component Score (PCS) and the Mental Component Score (MCS) (27).

### Statistical analyses

#### Association between infectious diseases and anxiety disorders

We used logistic regression analyses [odds ratio (OR) with 95% CI] to examine associations between infectious diseases (yes/no, the predictors in the model) and anxiety disorders (yes/no, the outcome in the model). We considered a *p* value <0.05 as statistically significant. As the analyses revealed that there was an association between both anxiety disorders and infectious diseases and sex the models were controlled for sex. We additionally built a variable called any anxiety disorder, which includes any of the anxiety disorders. As we had additional lifetime information for panic disorder and panic attacks, we checked for an association of these variables with infectious diseases.

We further tested whether subjects with one or more than one anxiety disorder report more infectious diseases than subjects without any anxiety disorder. As the assumptions (homoscedasticity and normality) were fulfilled, we set up a linear model with the number of anxiety disorders (three categories: none, one, and more than one disorder) as independent variable and the mean number of infectious diseases (ranging from zero to nine) as dependent variable. In all analyses, sex was included as covariate. For all analyses, we used the STATA software package, version 11.0 (28).

#### Association between comorbidity and quality of life

To analyze the association between anxiety disorders and infectious diseases and quality of life we used the two variables anxiety disorder (including any of the anxiety disorder) and infections (including any of the infectious diseases), both having two levels (yes/no). We combined these two variables and built a factor with four levels: one level with subjects with no infection and no anxiety disorder, one with only infection and no anxiety disorder, one with no infection but with anxiety disorder, and one level with both anxiety disorder and infection. To examine the association between this factor and the dependent variable, the PCS and MCS of the SF-36, we used a linear model with sex as covariate. For all analyses, we used the STATA software package, version 11.0 (28).

## Results

### Association of infectious diseases and anxiety disorders

As shown in Table 1, most associations (five out of nine) were found between whooping cough and 12-month anxiety disorders [ORs ranging from 1.52 (95% CI = 1.15–2.00) for simple phobia to 2.15 (95% CI = 1.36–3.40) for agoraphobia without panic disorder]. The associations between measles, rubella, chicken pox, and anxiety disorders were not significant. Additionally, most infectious diseases (4 out of 10) were associated with agoraphobia without panic disorder (ORs ranging from 1.79 (95% CI = 1.06–3.00) for mumps and 2.61 (95% CI = 1.001–6.82) for tuberculosis), whereas no associations between infectious diseases and phobic disorder not other specified, social phobia and obsessive–compulsive disorder were found.

**Table 1:** **Odds Ratios of infectious diseases (lifetime) for anxiety disorders (12 months) compared to the reference group that had no indexed anxiety disorder during the past 12 months (*N* = 4181)**.

DSM-IV mental disorder	Whooping cough (*n* = 977, 23.6%)	Measles (*n* = 2591, 63.2%)	Mumps (*n* = 2066, 48.7%)	Rubella (*n* = 1467, 35.1%)	Chicken pox (*n* = 2390, 58.1%)	Scarlet fever (*n* = 632, 14.9%)	Tuberculosis (*n* = 91, 2.2%)	Dysentery (*n* = 52, 0.9%)	Typhus (*n* = 48, 1.1%)	Diphtheria (*n* = 181, 4.1%)
Any anxiety disorder^a^ (*n* = 727, 14.5%)	1.69 (1.36–2.09)* (*n* = 220)	1.00 (0.78–1.28) (*n* = 458)	1.13 (0.92–1.40) (*n* = 377)	0.96 (0.78–1.20) (*n* = 265)	1.07 (0.84–1.36) (*n* = 430)	1.31 (1.02–1.68)* (*n* = 128)	1.51 (0.83–2.73) (*n* = 18)	1.90 (0.86–4.18) (*n* = 12)	1.17 (0.54–2.53) (*n* = 11)	1.79 (1.21–2.64)* (*n* = 49)
Panic disorder with/without agoraphobia (*n* = 121, 2.3%)	1.52 (0.97–2.34) (*n* = 41)	0.68 (0.41–1.13) (*n* = 73)	0.97 (0.62–1.52) (*n* = 65)	0.91 (0.58–1.44) (*n* = 46)	0.89 (0.54–1.45) (*n* = 72)	1.08 (0.65–1.80) (*n* = 25)	0.52 (0.07–3.87) (*n* = 1)	0.82 (0.19–3.48) (*n* = 2)	0.57 (0.07–4.31) (*n* = 1)	2.82 (1.48–5.39)* (*n* = 14)
Panic attack (*n* = 241, 4.7%)	1.49 (1.07–2.06)* (*n* = 76)	0.91 (0.62–1.34) (*n* = 149)	1.19 (0.85–1.66) (*n* = 130)	0.97 (0.69–1.35) (*n* = 89)	1.17 (0.79–1.72) (*n* = 149)	1.29 (0.88–1.89) (*n* = 47)	1.31 (0.52–3.34) (*n* = 6)	1.21 (0.33–4.35) (*n* = 4)	0.55 (0.13–2.36) (*n* = 2)	2.46 (1.46–4.14)* (*n* = 22)
Agoraphobia without panic disorder (*n* = 105, 2.0%)	2.15 (1.36–3.40)* (*n* = 42)	1.81 (0.91–3.60) (*n* = 81)	1.79 (1.06–3.00)* (*n* = 67)	1.12 (0.68–1.83) (*n* = 42)	0.78 (0.45–1.33) (*n* = 64)	1.97 (1.17–3.33)* (*n* = 25)	2.61 (1.001–6.82)* (*n* = 5)	2.71 (0.69–10.58) (*n* = 4)	2.12 (0.55–8.13) (*n* = 3)	1.93 (0.83–4.47) (*n* = 8)
Simple phobia (*n* = 388, 7.6%)	1.52 (1.15–2.00)* (*n* = 111)	0.80 (0.59–1.09) (*n* = 244)	1.00 (0.76–1.30) (*n* = 196)	1.03 (0.78–1.37) (*n* = 146)	1.18 (0.86–1.67) (*n* = 234)	1.18 (0.85–1.63) (*n* = 65)	1.54 (0.70–3.39) (*n* = 9)	1.15 (0.37–3.56) (*n* = 4)	0.96 (0.31–2.96) (*n* = 4)	1.50 (0.92–2.45) (*n* = 26)
Phobic disorder NOS (*n* = 173, 3.4%)	1.49 (0.99–2.24) (*n* = 48)	1.01 (0.63–1.61) (*n* = 110)	1.08 (0.73–1.60) (*n* = 88)	0.86 (0.58–1.28) (*n* = 61)	0.84 (0.55–1.28) (*n* = 102)	1.08 (0.66–1.76) (*n* = 26)	1.81 (0.57–5.73) (*n* = 5)	2.88 (0.76–10.82) (*n* = 3)	0.77 (0.17–3.38) (*n* = 2)	1.56 (0.76–3.17) (*n* = 10)
Social phobia (*n* = 94, 1.9%)	1.35 (0.80–2.27) (*n* = 26)	1.71 (0.85–3.45) (*n* = 55)	0.71 (0.43–1.18) (*n* = 45)	0.82 (0.49–1.38) (*n* = 33)	1.08 (0.56–2.10) (*n* = 55)	0.64 (0.31–1.34) (*n* = 10)	0.95 (0.21–4.17) (*n* = 2)	–^b^	0.24 (0.03–1.77) (*n* = 1)	1.36 (0.54–3.37) (*n* = 6)
Generalized anxiety disorder (*n* = 73, 1.5%)	1.94 (1.12 –3.35)* (*n* = 28)	1.28 (0.64–2.54) (*n* = 50)	1.28 (0.71–2.30) (*n* = 37)	0.80 (0.44–1.45) (*n* = 26)	1.40 (0.68–2.85) (*n* = 45)	1.32 (0.67–2.60) (*n* = 14)	1.41 (0.33–5.93) (*n* = 2)	1.62 (0.21–11.97) (*n* = 1)	–^b^	1.39 (0.31–6.53) (*n* = 2)
Obsessive–compulsive disorder (*n* = 38, 0.7%)	1.15 (0.53–2.49) (*n* = 11)	0.82 (0.35–1.94) (*n* = 25)	0.78 (0.34–1.76) (*n* = 16)	0.95 (0.41–2.20) (*n* = 13)	1.32 (0.51–3.38) (*n* = 23)	1.10 (0.36–3.27) (*n* = 5)	1.50 (0.32–6.80) (*n* = 2)	–^b^	–^b^	2.16 (0.62–7.54) (*n* = 3)

CI, confidence interval; DSM-IV, diagnostic and statistical manual of mental disorders, fourth edition; NOS, not other specified; OR, odds ratio; *n*, unweighted number of subjects, % weighted percentage, *p < 0.05, adjusted for sex.

*^a^including any anxiety disorder during the past 12 months*.

*^b^empty cell size*.

Whooping cough (OR = 1.69, 95% CI = 1.36–2.09), scarlet fever (OR = 1.31, 95% CI = 1.02–1.68), and diphtheria (OR = 1.79, 95% CI = 1.21–2.64) were associated with having any 12-month anxiety disorder.

Additional analyses with lifetime diagnoses of panic disorder and panic attacks and infectious diseases revealed that whooping cough (OR = 1.46, 95% CI = 1.02–2.07), typhus (OR = 4.24, 95% CI = 1.80–9.97), and diphtheria (OR = 2.39, 95% CI = 1.36–4.21) were associated with panic disorder, whereas whooping cough (OR = 1.42, 95% CI = 1.09–1.85), scarlet fever (OR = 1.37, 95% CI = 1.01–1.86), dysentery (OR = 2.41, 95% CI = 1.04–5.56), typhus (OR = 2.62, 95% CI 1.21–5.66), and diphtheria (OR = 2.25, 95% CI = 1.42–3.55) were associated with panic attacks during lifetime.

**Figure 1:**
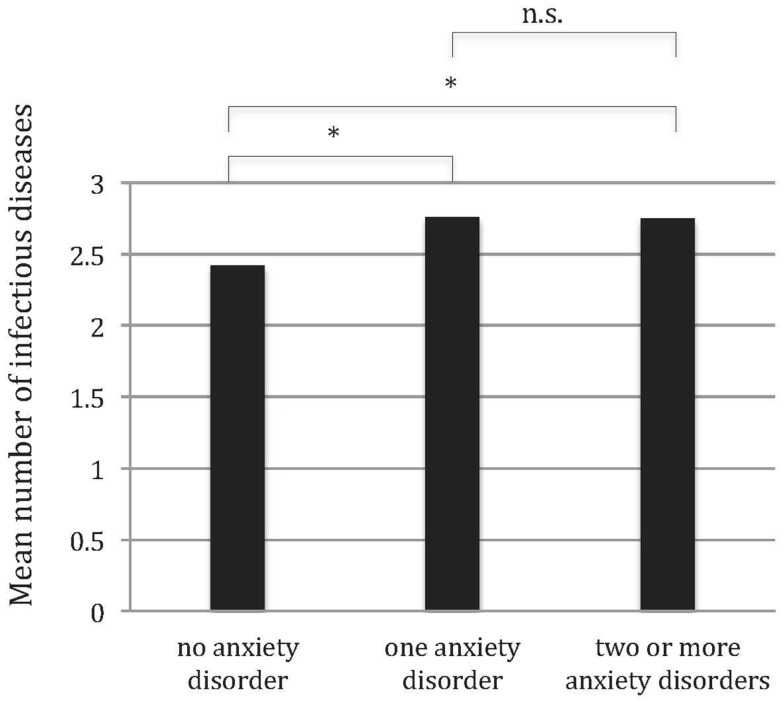
**Mean number of infectious diseases (lifetime) for subjects with no anxiety disorder, with one anxiety disorder, and with two or more anxiety disorders (12 months)**. Mean scores adjusted for sex; *n.s*. not significant, *p < 0.05, weighted data.

As shown in Figure 1, having one or more anxiety disorders was associated with an increased number of infectious diseases compared to no anxiety disorder (mean number of infectious diseases with no anxiety disorder: 2.42, 95% CI = 2.36–2.48; one anxiety disorder: 2.76; 95% CI = 2.60–2.91; two or more: 2.75; 95% CI = 2.51–2.99, *z* = 2.76, *p* < 0.006). Contrast analysis in addition showed that having more than one anxiety disorder did not further increase the number of infectious diseases relative to having one anxiety disorder (*z* = 0.26, *p* < 0.798).

### Association of comorbidity and quality of life

**Figure 2:**
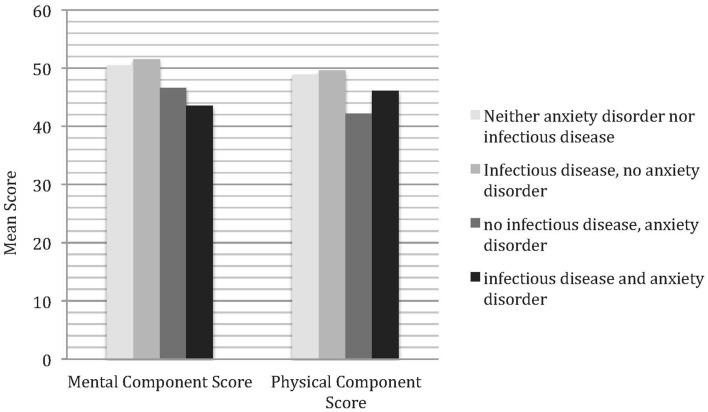
**The association of anxiety disorders and infectious diseases and health-related quality of life**. Mean scores adjusted for sex.

As shown in Figure 2, subjects with both infectious disease and anxiety disorders report a lower level in the MCS (*M* = 43.6, 95% CI = 42.5–44.7) than subjects with only infectious disease (51.6, 95% CI = 51.3–52.0, *z* = −14.23, *p* < 0.000) or neither of them (*M* = 50.5, 95% CI = 49.1–52.0, *z* = −7.32, *p* < 0.000). In the PCS subjects with only anxiety disorder (*M* = 42.3, 95% CI = 35.5–48.0, *z* = −2.09, *p* < 0.037) and with both infectious disease and anxiety disorder (*M* = 46.2, 95% CI = 45.2–47.1, *z* = −2.35, *p* < 0.019) report a lower level when compared to subjects with neither condition (*M* = 48.9, 95% CI = 47.2–50.7).

Additionally, subjects with only anxiety disorder (*M* = 42.3, 95% CI = 35.5–48.0) report a lower quality of life in the PCS compared to subjects with only infectious disease (*M* = 49.6, 95% CI = 49.2–50.0, *z* = −2.48, *p* < 0.013). Further, subjects with both anxiety disorder and infectious diseases (*M* = 46.2, 95% CI = 45.2–47.1) report a lower quality of life in the PCS when compared to subjects with only infectious disease (*M* = 49.6, 95% CI = 49.2–50.0, *z* = −6.61, *p* < 0.000). All other comparisons were not significant.

## Discussion

This study investigated the associations between infectious diseases and anxiety disorders in a representative adult community sample. The results of this study suggest that specific infectious diseases are associated with significantly increased prevalence of anxiety disorders, compared with those without infectious diseases, and that the co-occurrence of the two is associated with substantial loss in quality of life. Specifically, our findings revealed associations between whooping cough, scarlet fever, and diphtheria and increased likelihood of having anxiety disorder. Additionally, associations between whooping cough, mumps, scarlet fever, tuberculosis, dysentery, typhus, diphtheria, and specific anxiety disorders were found. Our findings are partly in line with an earlier study suggesting an association between an infection in the first year of life and panic disorder, social phobia, and overanxious disorder (14). As in that study, we found associations of infectious diseases and anxiety disorders in general. In contrast, we did not find an association between any of the investigated infectious diseases and social phobia.

The findings are based on data from a representative community sample and therefore the results are not limited by the same biases as in clinical samples. However, due to the cross-sectional nature of this study, no conclusions concerning the temporal sequence of infectious diseases and anxiety disorders can be made. Therefore to explain the observed associations, three different models will be considered.

First, infectious diseases may precede anxiety disorders. Through biochemical processes, infections may increase the risk of having an anxiety disorder. Some infectious diseases such as scarlet fever mainly have their first onset in early childhood (29), whereas most anxiety disorders emerge for the first time during puberty (17, 30). We might therefore suggest that some infectious diseases do emerge before anxiety disorders do. This interpretation may be supported by the findings of the above mentioned cross-sectional study addressing the relationship of infectious diseases and anxiety disorders, showing that severe infections during the first year of life (reported by the parents) are associated with increased anxiety disorders among 9–17-year olds, therefore suggesting that infections occur before anxiety disorders emerge (14). Research suggests that increased concentration of proinflammatory cytokines could contribute to feeling of depression, and also anxiety (13). Additionally, it is known that some viral infections, such as tuberculosis, can directly affect the brain (31). These processes may damage the brain and therefore cognitive impairments and behavioral changes can occur (31). Cognitive impairment in different domains such as executive functioning or visual memory has been found to be common among people with anxiety disorders (32) and is thought to reduce coping abilities and impact social and occupational functioning (32), which could lead to an intensification of anxiety symptoms.

Second, it may be that anxiety disorders precede infectious diseases. One explanation for this path would be that anxiety disorders are related to psychological disturbance and stress. Stress is associated with an increased cortisol secretion and may contribute to an immune function decline. An immune function decline in anxious subjects had been shown on a physiological level, namely in a reduction of chemotaxis, phagocytosis, and lymphoproliferation (33). Alternatively, studies have shown that the extent and quality of general medical health care among those with mental disorders might be poor (31). Even though the association has mainly been shown in psychotic and substance use disorders (31), it may be that people with anxiety disorders receive less medical information and therefore the frequency of vaccination could be lower among these subjects leading to increased rates of some infectious diseases.

Third, it may be that there is a common factor related to an increased risk of having both infectious diseases and anxiety disorders at the same time. Both genetic and environmental common factors could be considered. In our analyses, we found increased rates of whooping cough, scarlet fever, dysentery, typhus, and diphtheria in subjects with panic attacks. In prospective analyses, prior regular smoking was found to increase the risk of panic attacks (34). Smoking on the other hand increases the risk of bacterial or viral infections (35). One might therefore speculate that smoking could act as a common environmental risk factor for both infectious diseases and mental disorders.

Our results also show that individuals with both conditions have a loss in health-related quality of life. This is in line with earlier findings of those with anxiety disorders and chronic physical diseases reporting decreased health-related quality of life (15). Our analyses additionally show that infectious diseases are not associated with a significantly lower quality of life if they appear alone when compared to people with neither condition. In combination with anxiety disorders, however, they are. Our results therefore extend previous findings and show that not only chronic physical diseases, but – at least partly – well treatable and time-limited infections are related to lower quality of life in subjects with anxiety disorders.

The current study has a number of limitations. First, the prevalence rates of the infectious diseases are based on self-report. Therefore, it may be that some infectious diseases are misunderstood (e.g., whooping cough may be confused to cough in general or may be overreported). Even though there is evidence suggesting that the validity of self-assessment of certain infectious diseases such as a common cold is high (36), it is additionally known that negative emotional style can be associated with an over-reporting of unverified symptoms (reporting bias) (37). This needs to be addressed in future studies by assessing infectious diseases with medical interview or by laboratory blood tests. Second, the combination of infectious diseases and anxiety disorder led to some small cell sizes, especially in tuberculosis, dysentery, typhus, and diphtheria. Third, due to the cross-sectional nature of the study, no conclusions can be drawn concerning the causal relationship of the observed associations. Fourth, the results cannot be generalized to subjects younger than 18 and older than 65 years. Fifth, we only had 12-month diagnoses for anxiety disorders (except for panic disorder), whereas infectious diseases were assessed over lifetime. Future studies should address this issue by comparing both infectious diseases and anxiety disorders during the same time frame. Sixth, potential other confounders may play a role in the associations.

With these limitations in mind, our study extends prior findings (14) about the association between infectious diseases and anxiety disorders. Our study suggests that adults with infectious diseases report higher prevalence rates of anxiety disorders and that this comorbidity is associated with a health-related loss in quality of life. Future longitudinal studies should clarify the temporal relationship of these disorders and evaluate the pathways contributing to this comorbidity with targeted psychological and immunological parameters. The health-related loss in quality of life could underline the importance of disease prevention (e.g., vaccination) in people with anxiety disorders as especially subjects with both infectious disease and anxiety disorder reported an impairment in quality of life. The associations additionally show that infectious diseases and anxiety disorders might increase the risk of having one another, and/or they might have common shared risk factors at least in subtypes. This research could initiate future research on the linkage of infectious diseases and anxiety disorders.

## Conflict of Interest Statement

The authors declare that the research was conducted in the absence of any commercial or financial relationships that could be construed as a potential conflict of interest.
